# Reliability analysis of horseshoe tunnels by radial-based importance sampling method based on complex function displacement solution

**DOI:** 10.1371/journal.pone.0346030

**Published:** 2026-04-03

**Authors:** Yao Rong, Qiang Liu, Yang Sun, Xiangshen Chen, Xinwei Zhang, Songyan Li

**Affiliations:** 1 Jiangxi Provincial Key Laboratory of Highway Bridge and Tunnel Engineering and Jiangxi Communications Investment Maintenance Technology Group Co., Ltd., Nanchang, China; 2 State Key Laboratory of Safety and Resilience of Civil Engineering in Mountain Area, School of Civil Engineering and Architecture, East China Jiaotong University, Nanchang, China; 3 China Construction Fourth Engineering Bureau Co., Ltd, Guangzhou, China; University of Salerno: Universita degli Studi di Salerno, ITALY

## Abstract

Tunnel reliability analysis is an important analytical method to ensure tunnel safety. In this paper, the plane elastic complex variable function theory is adopted, and the explicit displacement function of the horseshoe-shaped tunnel vault is derived through conformal mapping. This function is verified by ABAQUS numerical analysis, and the correctness of the derivation is further confirmed by degenerating it to a circular tunnel. Subsequently, based on this explicit displacement function, a comparative analysis is conducted on four methods: the advanced first-order second moment (AFORM) method, Monte Carlo sampling (MCS) method, importance sampling (IS) method, and radial-based importance sampling (RBIS) method. The results show that the RBIS method has higher calculation accuracy and better efficiency. Finally, considering the influences of the mean value of load, load variation coefficient, and surrounding rock parameters, the reliability of the horseshoe-shaped tunnel and the circular tunnel is compared and analyzed based on the derived explicit function. It is found that except for the lateral pressure coefficient, the two types of tunnels are similar in terms of the sensitivity of other parameters and the influence laws of parameters on reliability. The research results can provide a reference for the analogical design and analysis of horseshoe-shaped tunnels and circular tunnels.

## 1. Introduction

Tunnel engineering is widely applied in infrastructure fields such as transportation and water conservancy, and its structural safety is of vital importance to project operation. The analysis of tunnel stress and deformation is the core of safety assessment. Model tests and theoretical calculations are important methods for analyzing geotechnical engineering problems, among which theoretical calculations mainly include analytical methods and numerical methods [1–4]. Tunnel mechanics problems can be simplified to hole problems, but for non-circular tunnels, the displacement analytical solution is difficult to obtain directly. Researchers usually rely on the theory of plane elastic complex variable function, transforming non-circular problems into circular ones through conformal mapping to derive analytical solutions. For example, Kargar et al. [5] introduced the Cauchy solution in the complex function method to obtain the analytical solution of the stress in non-circular tunnels. Lu et al. [6] considered more complex hole shapes and took straight wall semi-circular arch tunnel as an example to obtain the analytical solution of the non-circular tunnel. Guan et al. [7] proposed the displacement back analysis technique of mountain tunnel using the theory of plane elastic complex variable function. Zeng et al. [8] deduced the analytical solution of displacement and stress caused by non-circular tunnel excavation considering viscoelastic constitutive. These studies have laid a solid theoretical foundation for the mechanical analysis of non-circular tunnels.

However, in practical engineering, geological parameters, load conditions and other factors have inherent randomness, making uncertainty analysis indispensable for tunnel safety evaluation [9–11]. Reliability analysis methods are the key technical means to solve such problems, which are mainly divided into approximate analytical methods, digital simulation methods and surrogate model methods. Approximate analytical methods include Rosenblueth point estimation method, first-order second-moment method, second-order second-moment method and higher-order second-moment method [12,13]; digital simulation methods are represented by Monte Carlo Sampling (MCS) method, Importance Sampling (IS) method and Subset Simulation (SS) method [14,15]. For complex engineering problems where explicit functional functions are difficult to establish, surrogate model methods can construct implicit functions, and common models include Polynomial Response Surface Method (PRSM), Radial Basis Function (RBF) model, Kriging model, Support Vector Regression (SVR) model, Artificial Neural Network (ANN) model and Moving Least Square Method (MLSM) surrogate model [16,17].

In recent years, New reliability analysis methods are being continuously proposed, tunnel engineering reliability has attracted extensive attention from scholars [18–22]. Napa-Garcia et al. [23] used three point estimation methods to analyze the reliability of shallow circular tunnels and verified the accuracy of different methods; Guo et al. [24] focused on the reliability of excavation support for shallow circular tunnels and discussed the influence of spatial variability and autocorrelation of soil on lining reliability; Lu et al. [25] analyzed the spatial variability of tunnel rock mass properties by using interpolated autocorrelation combined with finite difference analysis. Zhou et al. [26] adopted the Active Learning-Kriging-Monte Carlo Simulation (AK-MCS) method to study the impact of weak interlayers on the stability of roadway working faces. Although these studies have enriched the research system of tunnel reliability, obvious limitations still exist: most reliability analyses based on tunnel analytical solutions are limited to circular tunnels, while non-circular tunnels mostly rely on surrogate model methods. As a typical non-circular tunnel widely used in engineering, the uncertainty analysis of horseshoe tunnels based on displacement analytical solutions has not been reported yet. This research gap makes it difficult to directly apply accurate analytical solutions to the reliability evaluation of horseshoe tunnels, affecting the scientificity of engineering design and safety assessment.

To fill this gap, this study focuses on the reliability analysis of horseshoe tunnels based on displacement analytical solutions. First, based on the theory of plane elastic complex variable function, considering the support pressure and verifying with existing results, the analytical expression of vault settlement of non-circular tunnels is derived, and on this basis, the explicit functional function for reliability analysis of horseshoe tunnels is established. Second, to select an efficient and accurate reliability calculation method, the performance of Advanced First-Order Second-Moment (AFORM) method, MCS method, IS method and Radial-Based Importance Sampling (RBIS) method is compared and analyzed, and the RBIS method with better comprehensive performance is determined. Finally, using the RBIS method, the influences of load mean value, surrounding rock parameters, load variability and the interaction of surrounding rock parameters on the reliability of horseshoe tunnels are systematically analyzed. The research results are expected to provide a new theoretical method for the reliability evaluation of horseshoe tunnels and technical support for the optimization of engineering design and the improvement of safety guarantee level.

## 2. Functional function derivation

In order to derive the analytic explicit functional function of horseshoe tunnel, it is necessary to derive the elastic displacement solution of tunnel vault with the complex function theory and then establish the function using the ultimate displacement theory.

### 2.1. Computational model and basic theory

**Fig 1:**
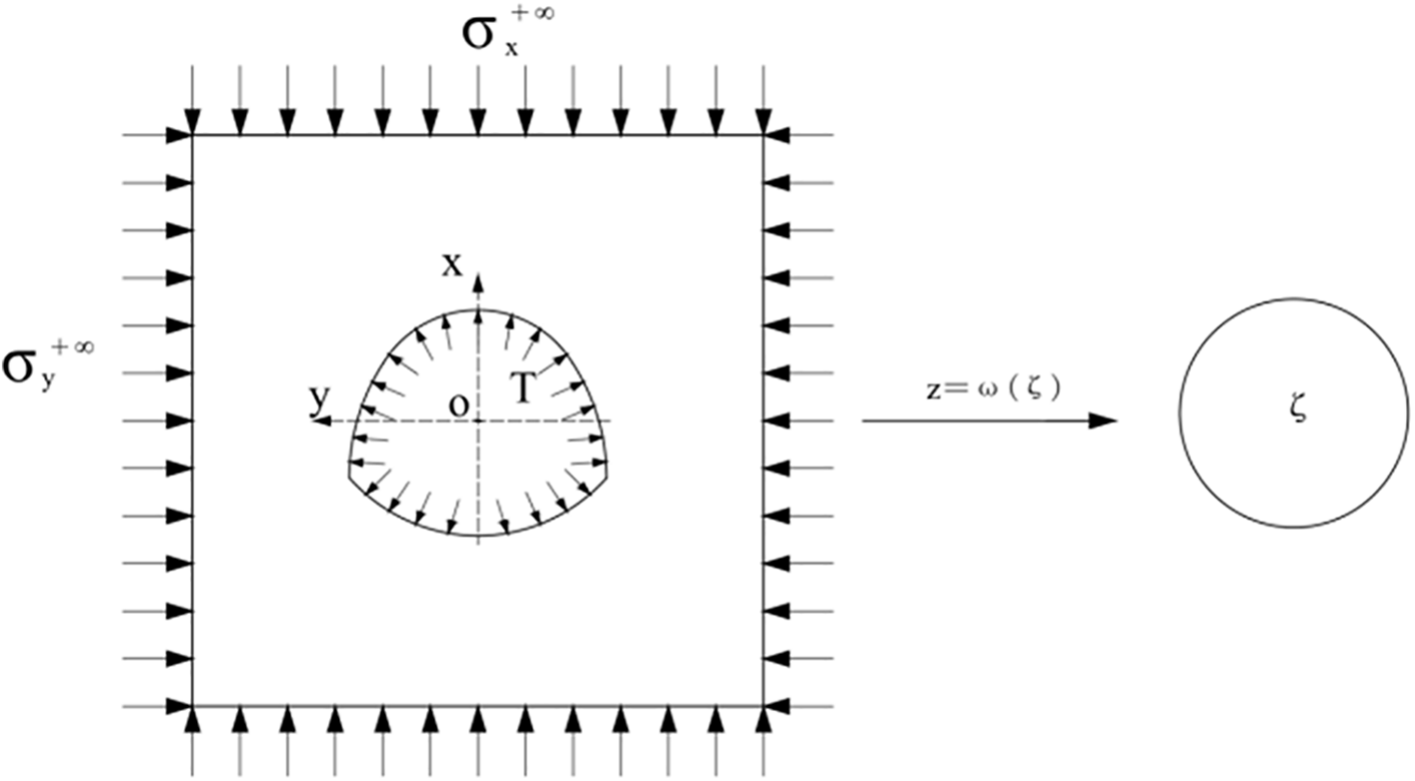
Schematic diagram of the analysis model and conformal mapping.

Because the gravity gradient's influence cannot be considered when the buried depth and aperture of the tunnel are large, the surrounding rock around the tunnel can be regarded as an external load affected by the far-field stress. Section tunnel excavation of homogeneous surrounding rock can be simplified as a plane strain problem. In this paper, the curved wall horseshoe tunnel excavation was taken as an example. The calculation diagram is shown in Fig 1 and $\overset{+\infty }{\underset{x}{\sigma }}\text{ and }\overset{+\infty }{\underset{y}{\sigma }}$ represents the vertical and horizontal ground stress of original rocks at infinity, and the lining support function is simplified as the *X* uniform pressure *T* applied on the excavation inner contour surface.

According to the theory of plane elasticity and complex variable function, the hole on the *Z* plane can be transformed into the unit circle on the ζ*ζ* plane through the conformal mapping function Z=ω(ζ). The conformal maps can generally be written in the form of power series.


$$Z=\omega (\zeta )=R\sum_{k=\text{0}}^{n}\left[\zeta +C_{k}\zeta^{-k}\right]$$ (1)

where *R* represents the hole size; $C_{k}$ characterizes the shape of the hole, *k* = 1, 2,..., n, a complex number. If the cavity is symmetrical, it will degenerate into a real number. These parameters can be obtained by optimization-based methods, and Many researchers have contributed their works to this issue [6]. Generally, we take n = 5 in this article for the sake of convenience.

According to the classical theory of elasticity [27], the stress and displacement distribution on the plane (polar coordinate system) and the original plane (rectangular coordinate system) can be expressed by two analytic functions ϕ(ζ) and ψ(ζ). According to the boundary condition Eq. (4), φ(ζ)ϕ(ζ) and ψ(ζ)ψ(ζ) can be obtained following Eq. (2),(3). Finally, the displacement can be solved by Eq. (5).


$$\phi (\zeta )=-\frac{\text{1}}{\text{2}\pi (\text{1}+k)}\left(\overset{―}{F_{x}}+i\overset{―}{F_{x}}\right)ln\zeta +(B+iC)\omega (\zeta )+\phi_{\text{0}}(\zeta )$$ (2)

$$\psi (\zeta )=\frac{\text{1}}{\text{2}\pi (\text{1}+k)}\left(\overset{―}{F_{x}}-i\overset{―}{F_{x}}\right)ln\zeta +\left(B^{\prime }+iC^{\prime }\right)\omega (\zeta )+\psi_{\text{0}}(\zeta )$$ (3)

$$\phi (\zeta )+\frac{\omega (\zeta )}{\overset{―}{\omega^{\prime }(\zeta )}}\overset{―}{\omega^{\prime }(\zeta )}+\psi (\zeta )=f(\zeta )$$ (4)

$$\text{2}G\left(\mu_{x}+i\mu_{y}\right)=\text{2}G\left(\mu_{\rho }+i\mu_{\theta }\right)e^{i\alpha }=k\phi (\zeta )\frac{\omega (\zeta )}{\overset{―}{\omega^{\prime }(\zeta )}}\overset{―}{\phi^{\prime }(\zeta )}-\psi (\zeta )$$ (5)

where α is the angle between the two sets of coordinate systems for the same point;$\;\mu_{x}$ and $\mu_{y}$ are the vertical and horizontal displacement on the original plane; $\mu_{\rho }$ and $\mu_{\theta }$ are radial displacement and circumferential displacement on the image plane. $B=\frac{\overset{+\infty }{\underset{x}{\overset{+\infty }{\underset{y}{\sigma }}+\sigma }}}{\text{4}}$; $B^{\prime }=\frac{\overset{+\infty }{\underset{x}{\overset{+\infty }{\underset{y}{\sigma }}-\sigma }}}{\text{4}}$; $C^{\prime }=\overset{+\infty }{\underset{xy}{\tau }}$. For the plane strain problem, $G=\frac{E}{\text{2}+\text{2}\mu }$; k=3−4μ*.*

### 2.2. Derivation of analytical solution of displacement

Due to the compressive stress at infinity, the inner boundary of the tunnel is not subjected to force, so the force $\overset{―}{F_{x}}$ and $\overset{―}{F_{y}}$ are 0; and C can be taken as 0. The analytic function Eq. (2),(3) can be rewritten as the power series of Eq. (6),(7), respectively.


$$\phi (\zeta )=\frac{\overset{+\infty }{\underset{x}{\overset{+\infty }{\underset{y}{\sigma }}+\sigma }}}{\text{4}}\omega (\zeta )+\sum_{k=\text{1}}^{n}a_{k}\zeta^{-k}$$ (6)

$$\psi (\zeta )=(\frac{\overset{+\infty }{\underset{x}{\overset{+\infty }{\underset{y}{\sigma }}-\sigma }}}{\text{4}}+i\overset{+\infty }{\underset{xy}{\tau }})\omega (\zeta )+\sum_{k=\text{1}}^{n}b_{k}\zeta^{-k}$$ (7)

where $a_{k}$, $b_{k}$ are both coefficients.

Assuming that the internal boundary acts on the uniform radial pressure *T* provided by the lining support, so the stress boundary condition is as follow:


$$\phi_{\text{0}}(\zeta )+\frac{\omega (\zeta )}{\overset{―}{\omega^{\prime }(\zeta )}}\overset{―}{\overset{\prime }{\underset{\text{0}}{\omega }}(\zeta )}+\psi_{\text{0}}(\zeta )=t=T\omega (\gamma )-T\omega \left(\gamma_{\text{0}}\right)-\text{2}B\omega (\gamma )-(B^{\prime }-iC^{\prime })\overset{―}{\omega (\gamma )}$$ (8)

where $\gamma_{\text{0}}$ is starting point of the boundary.

Using the Harnack theorem, the Cauchy integral is carried out for stress boundary condition Eq. (8). By using the characteristics of unit circle boundary Cauchy integrals, the analytic function $\phi_{\text{0}}(\zeta )$ can be rewritten as:


$$\phi_{\text{0}}(\zeta )=\frac{\text{1}}{\text{2}\pi i}\oint \frac{\omega (\gamma )}{\overset{―}{\omega^{\prime }(\gamma )}}\frac{\overset{―}{\phi_{\text{0}}(\gamma )}}{\gamma -\zeta }d\gamma -\frac{\text{1}}{\text{2}\pi i}\oint \frac{T\omega (\gamma )-\text{2}B\omega (\gamma )-{(B}^{\prime }-iC^{\prime })\overset{―}{\omega (\gamma )}}{\gamma -\zeta }d\gamma$$ (9)

Substituting Eq. (1),(6) into the above equation and conducting Cauchy integral along the unit circle, the first term in the right-hand side of Eq. (9) can be rewritten as:


$$\frac{\text{1}}{\text{2}\pi i}\oint \frac{\omega (\gamma )}{\overset{―}{\omega^{\prime }(\gamma )}}\frac{\overset{―}{\phi_{\text{0}}(\gamma )}}{\gamma -\zeta }d\gamma =-\sum_{k=\text{1}}^{n}S_{k}\zeta^{-k}$$ (10)

where,


$$\left\{ \begin{array}{c} S_{\text{1}}=-\text{3}L_{\text{5}}\overset{―}{a_{\text{3}}}-\text{2}L_{\text{4}}\overset{―}{a_{\text{2}}}-L_{\text{3}}\overset{―}{a_{\text{1}}},\;L_{\text{3}}=C_{\text{3}}+C_{\text{1}}C_{\text{5}}\\ S_{\text{2}}=-\text{2}L_{\text{5}}\overset{―}{a_{\text{2}}}-L_{\text{4}}\overset{―}{a_{\text{1}}},\;L_{\text{4}}=C_{\text{4}}\;\\ S_{\text{3}}=-L_{\text{5}}\overset{―}{a_{\text{1}}},\;L_{\text{5}}=C_{\text{5}}\; \end{array}\right.\;$$

where ${\text{S}}_{\text{k}}$ is temporary coefficients in the derivation process.

Similarly, the second term in the right-hand side of Eq. (9) can be rewritten as:


$$\frac{\text{1}}{\text{2}\pi i}\oint \frac{T\omega (\gamma )-\text{2}B\omega (\gamma )-{(B}^{\prime }-iC^{\prime })\overset{―}{\omega (\gamma )}}{\gamma -\zeta }d\gamma =(\text{2}B-T)R\sum_{k=\text{1}}^{\text{5}}C_{k}\zeta^{-k}+(B^{\prime }-iC^{\prime })R\zeta^{-\text{1}}$$ (11)

Merge Eq. (10,11), the final potential function $\phi_{\text{0}}(\zeta )$ expression is shown in Eq. (12). If there is no far-field shear stress $\overset{+\infty }{\underset{xy}{\tau }}$, and all coefficients $B^{\prime }=\text{0}$, and all ${\text{a}}_{\text{k}}$ in Eq. (12) are all real numbers.


$$\phi_{\text{0}}(\zeta )=\sum_{k=\text{1}}^{\text{5}}a_{k}\zeta^{-k}$$ (12)

where,


$$\left\{ \begin{array}{c} a_{\text{1}}=\text{3}L_{\text{5}}\overset{―}{a_{\text{3}}}+\text{2}L_{\text{4}}\overset{―}{a_{\text{2}}}+L_{\text{3}}\overset{―}{a_{\text{1}}}-\text{2}BRC_{\text{1}}-B^{\prime }R+iC^{\prime }R\\ a_{\text{2}}=\text{2}L_{\text{5}}\overset{―}{a_{\text{2}}}+L_{\text{4}}\overset{―}{a_{\text{1}}}-\text{2}BRC_{\text{2}}\;\\ a_{\text{3}}=L_{\text{5}}\overset{―}{a_{\text{1}}}-\text{2}BRC_{\text{3}}\;\\ a_{\text{4}}=-\text{2}BRC_{\text{4}}\;\\ a_{\text{5}}=-\text{2}BRC_{\text{5}}\; \end{array}\right.\;$$

Similarly, by conjugating both ends of stress boundary condition Eq. (8), using Harnack theorem and Cauchy integral of unit circle boundary, the analytic function $\psi_{\text{0}}(\zeta )$ can be rewritten as:


$$\psi_{\text{0}}(\zeta )=\frac{\text{1}}{\text{2}\pi i}\oint \frac{\overset{―}{\omega (\gamma )}}{\overset{―}{\omega^{\prime }(\gamma )}}\frac{\overset{\prime }{\underset{\text{0}}{\phi }}(\gamma )}{\gamma -\zeta }d\gamma -\frac{\text{1}}{\text{2}\pi i}\oint \frac{T\overset{―}{\omega (\gamma )}-\text{2}B\overset{―}{\omega (\gamma )}-{(B}^{\prime }+iC^{\prime })\omega (\gamma )}{\gamma -\zeta }d\gamma$$ (13)

Similarly, two terms on the right of Eq.(13) are expanded to power series and performed Cauchy integrals and then combined. Finally, the expression of potential function was obtained as follow:


$$\psi_{\text{0}}(\zeta )=\sum_{k=\text{1}}^{\text{3}}S_{k}\zeta^{k}-\frac{\overset{―}{\omega (\frac{\text{1}}{\zeta )}}}{\omega^{\prime }(\zeta )}\overset{\prime }{\underset{\text{0}}{\phi }}(\zeta )-TR\zeta^{-\text{1}}-\text{2}BR\zeta^{-\text{1}}-{(B}^{\prime }+iC^{\prime })R\sum_{k=\text{1}}^{\text{5}}C_{k}\zeta^{-k}$$ (14)

Substituting Eq. (12),Eq. (14) into Eq. (6),Eq. (7), the final expressions of two analytic function ϕ(ζ) and ψ(ζ) can be obtained. Substituting the final expression of two analytic functions ϕ(ζ) and ψ(ζ) into Eq. (5) and separate the real part and the imaginary part, the vertical and horizontal displacement distributions can be obtained. The whole calculation process in this part was realized by the MATLAB program.

### 2.3. Verification of analytical solution of displacement

**Fig 2:**
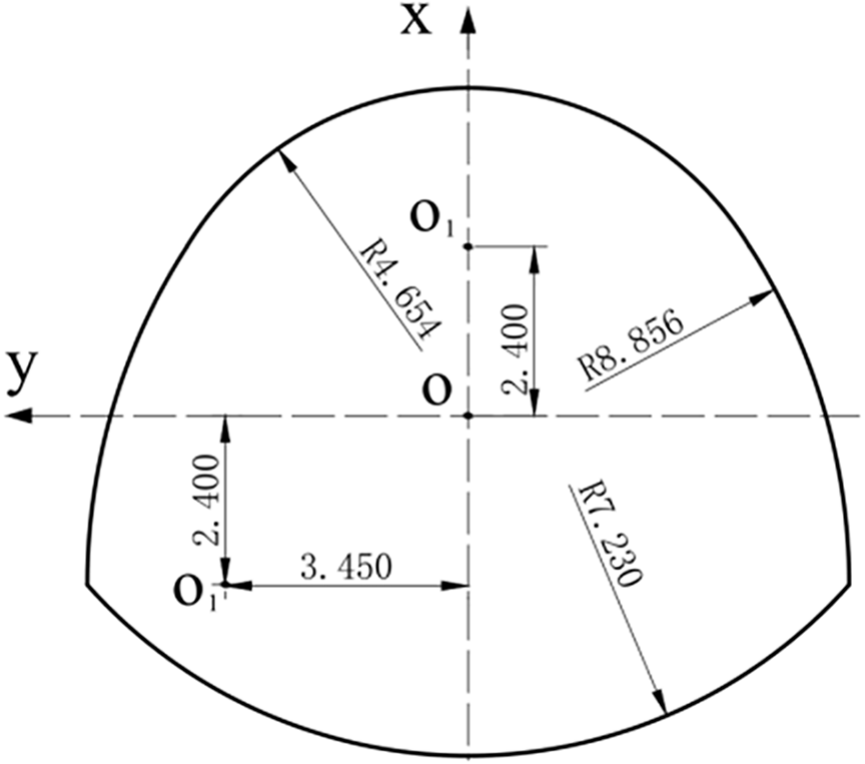
Cross-section of horseshoe tunnel.

In this paper, the following typical horseshoe tunnel was taken as an example, and the section size is shown in Fig 2.

According to the optimization method [7], the conformal mapping function can be solved as follow:


$$Z=\omega (\zeta )=\text{5}.\text{0}\text{3}\text{4}\text{9}(\zeta -\text{0}.\text{2}\text{0}\text{1}\text{9}-\text{0}.\text{1}\text{7}\text{7}\text{9}\zeta^{-\text{1}}+\text{0}.\text{2}\text{0}\text{1}\text{6}\zeta^{-\text{2}}-\text{0}.\text{0}\text{0}\text{8}\text{4}\zeta^{-\text{3}}-\text{0}.\text{0}\text{6}\text{3}\text{3}\zeta^{-\text{4}}+\text{0}.\text{0}\text{2}\text{2}\text{2}\zeta^{-\text{5}})$$

**Fig 3:**
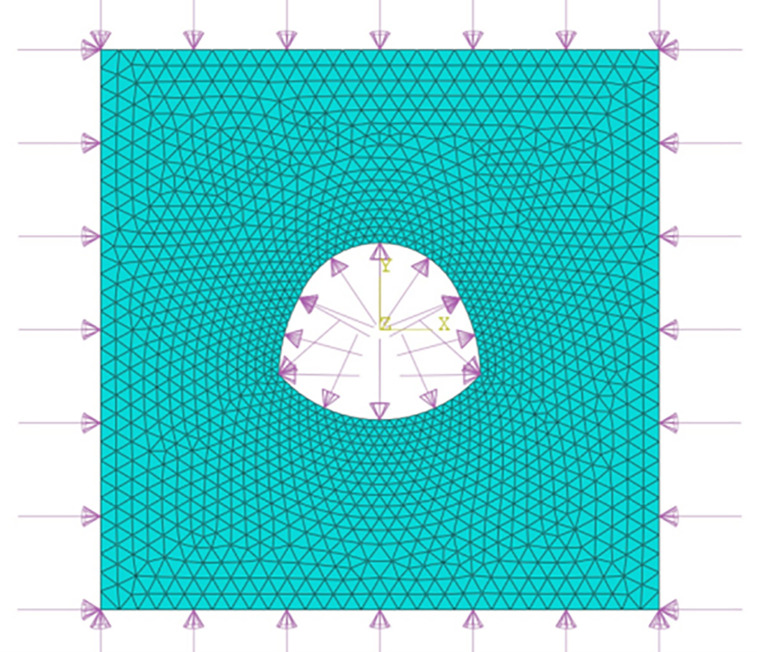
Finite element model diagram of horseshoe tunnel.

The displacement of the horseshoe tunnel was numerically calculated by ABAQUS software. The left and right boundaries of the model and the upper and lower boundaries are 30 m, and the calculation model is shown in Fig 3. The calculated parameters of the used elastic constitutive model are shown in Table 1.

**Table 1:** Calculation parameter values.

Parameter	*E*/MPa	*μ*	*P*/MPa	*λ*	𝐓/*T*/MPa
Value	10	0.3	2	1	0.1

Considering the vertical stress (*P*) and lateral pressure coefficient (λ), not considering the shear stress.

where


$$\left\{ \begin{array}{c} B=\frac{P(\text{1}+\lambda )}{\text{4}}{;B}^{\prime }=\frac{P(\lambda -\text{1})}{\text{2}}\\ \lambda =\frac{\overset{+\infty }{\underset{y}{\sigma }}}{\overset{+\infty }{\underset{x}{\sigma }}};C^{\prime }=\overset{+\infty }{\underset{xy}{\tau }}=\text{0} \end{array}\right.\;$$

**Fig 4:**
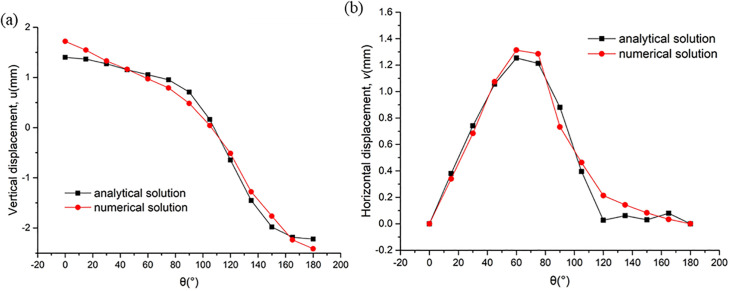
Analytical vs numerical solutions for vertical and horizontal displacements around a horseshoe tunnel.

To verify the calculation accuracy of the analytical solution derived in this paper for the surrounding displacement of the tunnel, observation points were arranged at intervals of 15° starting from the vault on the inner boundary of the tunnel. The vertical displacement and horizontal displacement of the excavation face of the horseshoe-shaped tunnel were solved using the numerical simulation method and the analytical method respectively, and a comparative analysis was conducted on the two types of results, as shown in Fig 4. The results indicate that: the variation trends of the analytical solution and the numerical solution are completely consistent; the vertical displacement shows a symmetric distribution along the 90° direction, while the horizontal displacement reaches its maximum at the 90° position, presenting an obvious asymmetric characteristic; meanwhile, the calculation results of the two solution methods are basically consistent, which fully proves that the analytical solution of the complex function displacement derived in this paper and the corresponding program are correct and reliable.

Based on this method, the analytical solution for the vertical displacement of the vault of a circular tunnel with radius *R* can be derived. When considering the effect of the supporting force *T*, the conformal mapping function of the circular tunnel is Z=ω(ζ)=Rζ. According to the MATLAB program compiled earlier, without considering the support force, the analytical solution for the vertical displacement of the vault is as follows:


$$U_{R}=\frac{\text{2}BR(\lambda -\text{1})\times (\mu +\mu^{\text{2}})}{E}+\frac{\left(-\text{2}T+\text{3}BR(\lambda -\text{1})-BP(\lambda +\text{1}))\times (\text{1}+\mu \right)}{\text{2}E}$$ (15)

According to the literature [28], the analytical solution of the vertical elastic displacement at any point (*r,θ*) in a circular tunnel in polar coordinates is


$$U_{R}=\frac{\left(\text{1}+\mu \right)}{E}\left\{-T\frac{R^{\text{2}}}{r}+\frac{(\text{1}+\lambda )PR^{\text{2}}}{\text{2}r}+\frac{(\lambda -\text{1})P\;cos\;\text{2}\theta }{\text{2}}\left[\text{4}(\text{1}-\mu )\frac{R^{\text{2}}}{r}-\frac{R^{\text{4}}}{r^{\text{3}}}\right]\right\}$$ (16)

On the inner surface of the tunnel vault, r=R, θ=0, it is found that the formula Eq. (15) is completely consistent with that of Eq. (16), which also proves that the derivation is correct.

### 2.4. Establishment of functional functions

According to reliability theory, the displacement of the tunnel supporting system is regarded as the generalized action effect, and the ultimate displacement is regarded as the generalized resistance. Obviously, all factors affecting the displacement are generalized random variables. So the deformation stability function of tunnel surrounding rock supporting system can be expressed as:


$$Z=g(R,S)=g(u_{R},u_{s})=u_{lim}-u_{max}\;$$

where R is the generalized resistance; $\mu_{lim}$ represents the limit position of surrounding rock; *S* is the generalized action effect; $\mu_{max}$ is the maximum displacement of a point in the surrounding rock (usually at the vaults or the waist of the walls).

In this paper, the elastic theory hypothesis was adopted, so the considered random variables were elastic modulus (*E*), Poisson's ratio (*μ*), vertical in-situ stress (*P*), lateral pressure coefficient (*λ*), and radial support force (*T*). The function of vault displacement judging the reliability of tunnel surrounding rock support system can be expressed as:


$$Z=g\left(\mu_{lim},\mu_{max}\right)=\mu_{lim}-\mu (X)=\mu_{lim}-\mu (E,\mu ,P,\lambda ,T)$$ (17)

Many scholars have done much research on the evaluation criteria of tunnel instability [29–31]. Some studies have used the magnitudes of surrounding rock displacement, stress, and plastic zone as thresholds for reliability calculation, and the coupled consideration of displacement, stress, and plastic zone is more valuable for truly reflecting tunnel reliability. This paper only aims to illustrate that the coupling of tunnel analytical methods with reliability can improve the efficiency of tunnel reliability calculation; therefore, the classic Prandtl theory for tunnels is adopted herein solely to calculate displacement, and a performance function is established to realize the rapid prediction of tunnel reliability. Proposed by Russian scholars based on empirical statistics, the Prandtl theory is suitable for tunnels with low surrounding rock strength. This method treats the rock mass as a homogeneous granular material, ignoring structural characteristics such as bedding and joints, and serves as a simple, rapid, and conventional method for calculating the deformation of tunnel surrounding rock. The formula for calculating the ultimate displacement of vault settlement is as follows:


$$\mu_{lim}=\delta_{top}=\text{1}\text{2}\frac{b_{\text{0}}}{f^{\text{1}.\text{5}}}$$ (18)

where f is the Platt coefficient of the rock; $b_{\text{0}}$ is the cavern span.

Take the example in Section 2, the span of the horseshoe tunnel is $b_{\text{0}}=\text{1}\text{0}.\text{1}\text{4}\text{9}$*,* f=20, and $\mu_{lim}=\text{1}.\text{4}\text{5}\text{0}\text{4}\text{mm}$ is calculated by Eq. (18).

By solving the displacement analytical solution of the above complex variable function, E,μ,P,λ,T are taken as variables to derive the analytical expression of vertical settlement displacement of horseshoe tunnel arch. According to Eq. (17), the display function of the vault settlement of horseshoe tunnel is


$$g=\text{1}.\text{4}\text{5}\text{0}\text{4}-\frac{\left(\left(\text{9}.\text{6}\text{0}\text{2}\text{4}P(\lambda -\text{1})-\text{0}.\text{3}\text{4}\text{4}\text{2}P(\lambda +\text{1})\right)\times \left(\mu +\mu^{\text{2}}\right)\right)}{E}-\frac{\left(\left(-T+\text{7}.\text{1}\text{4}\text{9}\text{9}P(\lambda -\text{1})-\text{2}.\text{7}\text{9}\text{6}\text{7}P(\lambda +\text{1})\right)\times (\text{1}+\mu )\right)}{E}$$ (19)

## 3. Reliability analysis method

### 3.1. The advanced first-order second moment method (AFORM)

AFORM is used to select the function's linearised points on the failure surface as checking points while considering the basic random variables’ actual distribution [32]. For a given nonlinear function, the potential failure point cannot be assured in advance, so it is necessary to continuously iterate to obtain a more accurate design checking point. The following is the basic principle of AFORM.

Set the design checking point in the failure domain as $P^{*}(\overset{*}{\underset{\text{1}}{x}},\;\overset{*}{\underset{\text{2}}{x}},\;\cdots ,\;\overset{*}{\underset{n}{x}})$ and expand the nonlinear functional function at the design checking point to take the linear part into account, as follow:


$$Z=g\left(X_{\text{1}},X_{\text{2}},\cdots ,X_{n}\right)\approx g\left(\overset{*}{\underset{\text{1}}{x}},\;\overset{*}{\underset{\text{2}}{x}},\;\cdots ,\;\overset{*}{\underset{n}{x}}\right)+\sum_{i=\text{1}}^{n}{\left(\frac{\partial g}{\partial X_{i}}\right)}_{P^{*}}(X_{i}-\overset{*}{\underset{i}{x}})$$ (20)

Since $g\left(\overset{*}{\underset{\text{1}}{x}},\;\overset{*}{\underset{\text{2}}{x}},\;\cdots ,\;\overset{*}{\underset{n}{x}}\right)=\text{0},\;$ it can be substituted into the above equation Eq. (20) to obtain:


$$Z\approx \sum_{i=\text{1}}^{n}{\left(\frac{\partial g}{\partial X_{i}}\right)}_{P^{*}}(X_{i}-\overset{*}{\underset{i}{x}})$$ (21)

The reliability index of the functional function is derived in Eq. (22), and the failure probability can be calculated by Eq. (23).


$$\beta =\frac{\sum_{i=\text{1}}^{n}{\left(\frac{\partial g}{\partial X_{i}}\right)}_{P^{*}}(\mu_{X_{i}}-\overset{*}{\underset{i}{x}})}{\sqrt{\sum_{i=\text{1}}^{n}{\left({\left(\frac{\partial g}{\partial X_{i}}\right)}_{P^{*}}\right)}^{\text{2}}}\overset{\text{2}}{\underset{X_{i}}{\sigma }}}$$ (22)

$$P_{f}=\emptyset (-\beta )$$ (23)

### 3.2. Montecarlo sampling method (MCS)

The basic idea of the MCS method is to estimate the failure probability by the ratio of the number of sample points falling into the failure domain to the total number of projection points [33]. The exact expression of failure considers the integral of the joint probability density function of input variables in the failure domain, which can be rewritten as the mathematical expectation form of the failure domain indicator function $I_{F}(x)$, namely:


$$P_{f}=\int_{F}f_{X}(x)d_{x}=\int_{R^{n}}I_{F}(x)f_{X}(x)d_{x}=E\left[I_{F}(x)\right]$$ (24)

Where R is an n-dimensional vector space, and ${\text{f}}_{\text{x}}(\text{x})$ is the joint probability density function of the fundamental random variable.

Eq. (24) indicates that structural failure probability is the failure domain indicator's mathematical expectation. According to the law of large numbers, the failure domain indicator function's mathematical expectation can be approximated by the sample mean of the failure domain indicator function. Therefore, the estimated value of failure probability can be expressed in Eq. (25) below:


$$\hat{P_{f}}=\frac{\text{1}}{N}\sum_{j=\text{1}}^{N}I_{F}\left(x_{j}\right)=\frac{N_{f}}{N}$$ (25)

The variance $\text{Var}\left[\hat{P_{f}}\right]$ of the estimated failure probability $\hat{P_{f}}$ and the estimated coefficient of variation $Cov\hat{P_{f}}$ are:


$$Var\left[\hat{P_{f}}\right]\approx \frac{\hat{P_{f}}-{\hat{P_{f}}}^{\text{2}}}{N-\text{1}}$$ (26)

$$Cov\hat{\left[P_{f}\right]}\approx \frac{\sqrt{Var\left[\hat{P_{f}}\right]}}{E\hat{\left[P_{f}\right]}}\approx \sqrt{\frac{\text{1}-\hat{P_{f}}}{(N-\text{1})\hat{P_{f}}}}$$ (27)

### 3.3. Important Sampling method(IS)

The basic idea of the IS method is to reduce the variance and improve the calculation accuracy by changing the probability distribution of the existing sample space and keeping the mathematical expectation unchanged to achieve the purpose of shortening the calculation time under the same precision requirements [34].

IS method converts the integral formula of failure probability to the form of mathematical expectation of its density function by introducing sampling density function:


$$\hat{P_{f}}=\int_{R^{n}}I_{F}(x)f_{X}(x)dx=\int_{R^{n}}I_{F}(x)\frac{f_{X}(x)}{h_{X}(x)}h_{x}dx=E\left[I_{F}\frac{f_{X}(x)}{h_{X}(x)}\right]$$ (28)

Where *R* is n-dimensional vector space, and $f_{X}(x)$ is the joint probability density function of the fundamental random variable. According to the sampling density function $h_{X}(x)$, sampling *N* sample points, the estimated failure probability of Eq. (28) is:


$$\hat{P_{f}}=\frac{\text{1}}{N}\sum_{j=\text{1}}^{N}\left[I_{F}(x_{j})\frac{f_{X}(x_{j})}{h_{X}(x_{j})}\right]$$ (29)

Similarly,


$$Var\left[\hat{P_{f}}\right]\approx \frac{\text{1}}{N-\text{1}}\left\{\frac{\text{1}}{N}\sum_{j=\text{1}}^{N}\left[I_{F}(x_{j})\frac{\overset{\text{2}}{\underset{X}{f}}(x_{j})}{\overset{\text{2}}{\underset{x}{h}}(x_{j})}\right]-{\hat{P_{f}}}^{\text{2}}\right\}$$ (30)

$$Cov\hat{\left[P_{f}\right]}\approx \frac{\sqrt{Var\left[\hat{P_{f}}\right]}}{E\left[\hat{P_{f}}\right]}$$ (31)

The design checking points of the function are calculated by AFORM and the importance of sampling density function $h_{X}(x)$ is constructed by setting the design checking point as the sampling centre. The failure probability is estimated by Eq. (29), and the corresponding coefficient of variation is obtained by Eq. (30,31).

### 3.4. The radial-based importance sampling method (RBIS)

**Fig 5:**
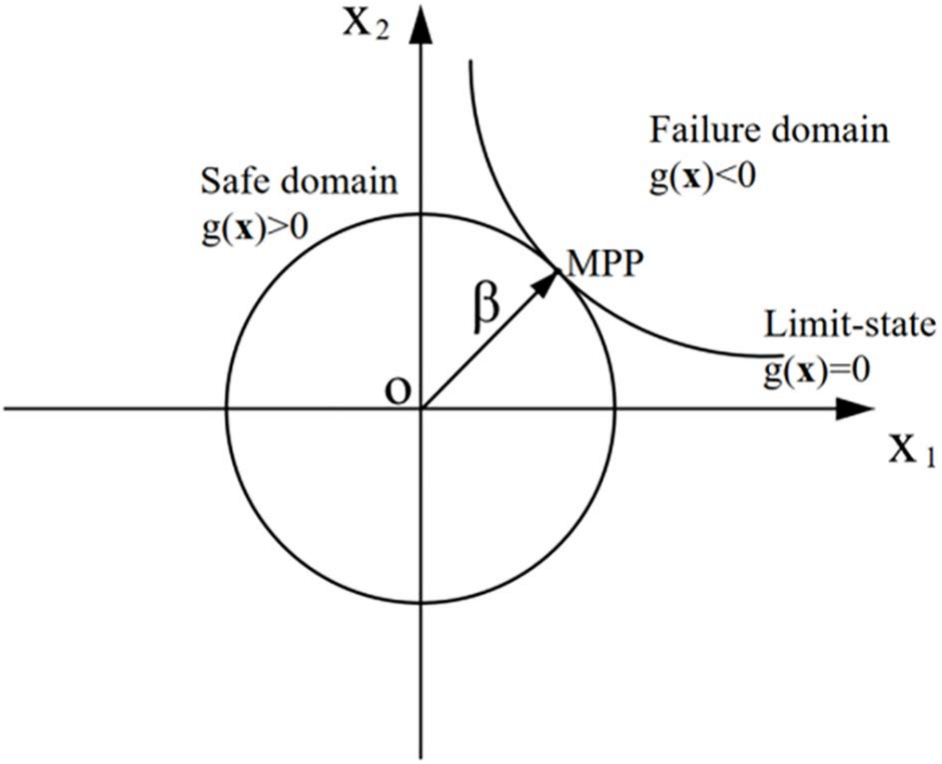
Schematic diagram of β sphere in two-dimensional case.

RBIS method [35–37] is developed based on the IS method. In the standard normal space, the hypersphere with the origin as the centre of the sphere and the distance between the origin and the design checking point as the radius is introduced, which is called β-hypersphere. The sampling is controlled outside the β-hypersphere. Based on the traditional sampling method, the sampling in the structural safety domain is further reduced, so the sampling efficiency is improved [34]. The β-hypersphere divides the space $R^{n}$ into ${\left\|x\right\|}^{\text{2}}<\beta^{\text{2}}$ and ${\left\|x\right\|}^{\text{2}}\geq \beta^{\text{2}}$, The optimal radius β is the shortest distance from the coordinate origin to the limit state surface, and the intersection point with the limit state surface is the most probable failure point (MPP). So the area determined by ${\left\|x\right\|}^{\text{2}}<\beta^{\text{2}}$ must be in the security domain. The schematic under a two-dimensional ball is shown in Fig 5.

The indicator function $I_{\beta }(x)$ in the outer region of β-hypersphere is defined as


$$I_{\beta }(x)=\left\{ \begin{array}{c} \text{1},{\left\|x\right\|}^{\text{2}}\geq \beta^{\text{2}}\\ \text{0},{\left\|x\right\|}^{\text{2}}<\beta^{\text{2}} \end{array}\right.\;$$

The probability density function of important sampling truncated by β-hypersphere; namely, the probability density function of truncated important sampling $\overset{tr}{\underset{X}{h}}(x)$ is:


$$\overset{tr}{\underset{X}{h}}(x)=\left\{ \begin{array}{c} \frac{h_{X}(x)}{\int_{R^{n}}I_{\beta }(x)h_{X}(x)dx},{\left\|x\right\|}^{\text{2}}\geq \beta^{\text{2}}\\ \text{0},{\left\|x\right\|}^{\text{2}}<\beta^{\text{2}} \end{array}\right.\;$$

According to the sampling failure probability formula, the calculation formula of truncated sampling failure probability can be deduced as follow:


$$P_{f}=\int_{R^{n}}I_{F}(x)I_{\beta }(x)\frac{f_{X}(x)}{h_{X}(x)}h_{X}(x)dx=E\left[I_{F}(x)I_{\beta }(x)\frac{f_{X}(x)}{h_{X}(x)}\right]$$ (32)

Similarly,


$$\hat{P_{f}}=\frac{\text{1}}{N}\sum_{j=\text{1}}^{N}\left[I_{F}(x_{j})I_{\beta }(x_{j})\frac{f_{X}\left(x_{j}\right)}{h_{X}\left(x_{j}\right)}\right]$$ (33)

Similarly,


$$Var\left[\hat{P_{f}}\right]\approx \frac{\text{1}}{N-\text{1}}\left\{\frac{\text{1}}{N}\sum_{j=\text{1}}^{N}\left[I_{F}(x_{j})I_{\beta }(x_{j})\frac{\overset{\text{2}}{\underset{X}{f}}(x)}{\overset{\text{2}}{\underset{X}{h}}(x)}\right]-{\hat{P_{f}}}^{\text{2}}\right\}$$ (34)

$$Cov\hat{\left[P_{f}\right]}=\frac{\sqrt{Var\left[\hat{P_{f}}\right]}}{E\hat{\left[P_{f}\right]}}$$ (35)

The important steps to calculate β-hypersphere truncation are as follows:

Firstly, AFORM is used to calculate the design checking point of the function, and the IS density function $h_{X}(x)$ is constructed by setting the design checking point as the sampling centre; then, extract *N* samples of the input variable $X(x_{\text{1}},\;x_{\text{2}},\cdots ,\;x_{jn})$. According to $\overset{tr}{\underset{X}{h}}(x)$.

Secondly, the distance from each sample $x_{j}=(x_{j\text{1}},\;x_{j\text{2}},\cdots ,x_{jn})$ to the origin $\sum_{i=\text{1}}^{n}\overset{\text{2}}{\underset{ij}{x}}$ is calculated. If ${\left\|x\right\|}^{\text{2}}\geq \beta^{\text{2}}$, $X_{j}$ are sample points of $\overset{tr}{\underset{X}{h}}(x)$, $I_{\beta }\left(x_{j}\right)=\text{1}$, otherwise $I_{\beta }\left(x_{j}\right)=\text{0}$. If $I_{\beta }\left(x_{j}\right)=\text{1}$, calculate the corresponding function $\text{g}\left({\text{x}}_{\text{j}}\right)$ to determine the value of indicator functions $I_{F}\left(x_{j}\right)$*,* $I_{\beta }\left(x_{j}\right)$*,* $\frac{f_{X}(x_{j})}{h_{X}(x_{j})}$, and $I_{F}\left(x_{j}\right)$*,* $I_{\beta }\left(x_{j}\right)$*,* $\frac{f_{X}(x_{j})}{h_{X}(x_{j})}$ are cumulated.

Finally, the failure probability and the corresponding coefficient of variation is obtained by Eq. (33–35).

### 3.5. Method comparison

In order to compare and analyze the four reliability methods above, the horseshoe tunnel display function Eq. (19) obtained in Section 3.1 was adopted. Five parameters were taken as random variables, including E,μ,P,λ,T. The selection of parameters refers to classic calculation examples from published relevant references. These examples are widely cited in the field of tunnel engineering reliability analysis and have good academic recognition and rationality. All parameters fall within the standard range of surrounding rock mechanical parameters. The setting of the surrounding rock lateral pressure coefficient takes into account the uniform distribution of surrounding rock stress in all directions, an assumption that is consistent with the stress and deformation state of the tunnel. Additionally, the probability distribution adopted in this study is the normal distribution [25], whose statistical characteristic parameters are presented in Table 2.

**Table 2:** Calculates parameter statistics.

Parameter	𝐄/E/MPa	μ	𝐏/P/MPa	λ	𝐓/T/MPa
Mean	15	0.3	2	1	0.2
Variation coefficient	0.12	0.22	0.24	0.1	0.1

These four reliability analysis methods were programmed in MATLAB, and the reliability calculated by the Monte Carlo method of 100 million times was selected as the exact solution. The comparative analysis was conducted in terms of sampling times $\left(N_{call}\right)$, reliability(β), failure probability$\left(P_{f}\right)$, coefficient of variation$\left(Cov\left[P_{f}\right]\right)$ and relative error ${(\varepsilon }_{\beta })$. The calculation results are shown in Table 3.

**Table 3:** Comparison of the calculation results of the four reliability methods.

Method	${𝐍}_{call}$	*β*	${𝐏}_{f}$	$𝐂𝐨𝐯[P_{f}]$	$\epsilon_{\beta }\%$
AFORM	\	2.0624	0.01958	\	0.71%
MCS	10^8^	2.0772	0.01889	0.00072	\
MCS	20000	2.0824	0.01865	0.05215	0.25%
IS	20000	2.0782	0.01876	0.01103	0.05%
RBIS	16875	2.0782	0.01876	0.01103	0.05%

To highlight the advantages of the RBIS method through comparative analysis, this study conducts an effective comparison with the results of the AFORM method, MCS, and IS method. The AFORM method exhibits a relatively large relative error. A comparison of 20,000 sample sets from the MCS and IS methods reveals that the IS method has a smaller coefficient of variation and relative error. When the reliability is the same, the sample size required by the RBIS method is significantly smaller than that of the IS method and the MCS method—with the sample size of the MCS method being three orders of magnitude larger. When the IS method and the MCS method use the same number of samples, it can be observed that the reliability error calculated by the IS method is three times that of the MCS method. Under the condition of the same coefficient of variation and relative error, the RBIS method not only requires fewer sampling times but also achieves higher computational efficiency due to the more targeted selection of samples. The test results fully demonstrate the advantages of the RBIS method. In the following analysis, the RBIS method will be used to discuss parameter sensitivity and the impact of parameter variability on reliability.

## 4. Results and discussion

To analyze the sensitivity and variation law of the relevant parameters of the horseshoe-shaped tunnel, the performance function of the horseshoe-shaped tunnel adopts Eq. (17), and the reliability analysis method uses RBIS. Take a circular tunnel with the same area as a comparison. The cross-section of the horseshoe tunnel adopts two sections, and the corresponding circular tunnel with the same area is a circular tunnel with an equal area of 4.997 radius. According to the same method in the second section above, the corresponding conformal mapping function is obtained as Z=ω(ζ)=4.997ζ*.* Then, the analytical solution of the vertical displacement at the circular arch is obtained based on the theory of the complex function of plane elasticity. According to the limit state equations Eq. (17),(18), the functional function of a circular tunnel can be deduced as follow:


$$g=\text{1}.\text{3}\text{4}\text{0}\text{8}-\frac{\text{9}.\text{9}\text{9}\text{4}P(\lambda -\text{1})\times \left(\mu +\mu^{\text{2}}\right)}{E}-\frac{\left(-T+\text{7}.\text{4}\text{9}\text{5}\text{5}P(\lambda -\text{1})-\text{2}.\text{4}\text{9}\text{8}\text{5}P(\lambda +\text{1}))\times (\text{1}+\mu \right)}{E}$$ (36)

The basic calculation parameters of circular tunnel and horseshoe tunnel are calculated according to the data in Table 2. During the discussion, a single variable is changing while other parameters remain unchanged.

### 4.1. Load analysis

This section mainly compares and analyzes the influence of mean changes of load parameters P,λ and T on the reliability of the horseshoe tunnel and circular tunnel.

**Fig 6:**
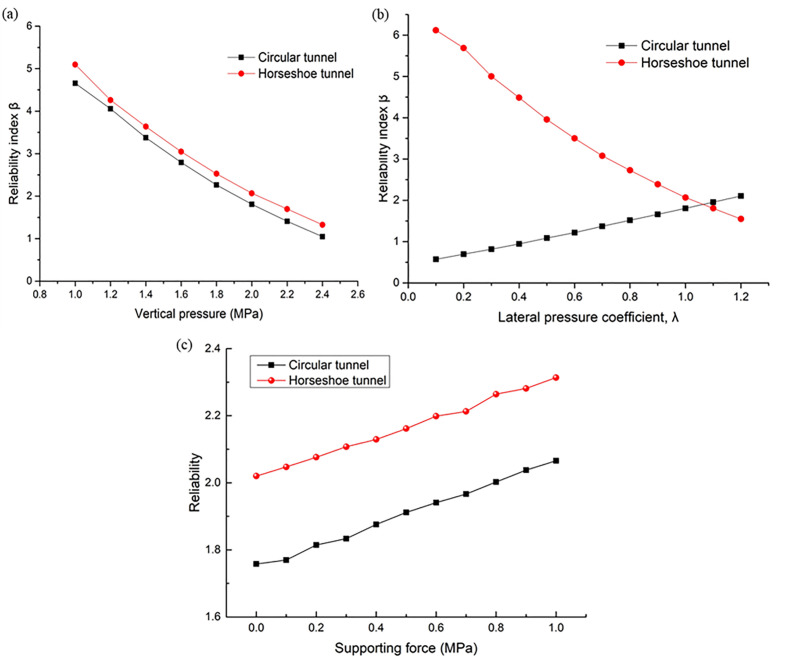
Influence of mean of load parameters on reliability.

In order to analyze the influence of mean P on the reliability of horseshoe tunnel and circular tunnel, controlled the average value of single variable P from 0.8 MPa to 2.6 MPa; the relationship curve between P and reliability is drawn as Fig 6 (a). It is found that the change law is the same, and both decrease with the increase of vertical pressure. Under the same pressure, the horseshoe tunnel vault displacement is smaller, so the horseshoe tunnel is slightly more reliable than a circular one. This is consistent with the qualitative analysis of vault displacement of the horseshoe and circular tunnels.

The λ is the ratio of horizontal pressure to vertical pressure. The mean value of the single variable λ is controlled to change uniformly from 0.1 to 1.2, the relationship curve between λ and reliability is shown in Fig 6 (b). By comparing circular tunnels and horseshoe-shaped tunnels, it is found that the reliability of horseshoe-shaped tunnels decreases with the increase of λ, while the reliability of circular tunnels increases with the increase of λ. The reason for this phenomenon is as follows: when λ is small, the vault of the circular tunnel is prone to be affected by vertical stress concentration, and the vault settlement is greater than that of the horseshoe-shaped tunnel, so the horseshoe-shaped tunnel has higher reliability; when λ is large, the symmetry of the circular cross-section is more compatible with the horizontal-dominant stress field, and the vault settlement is smaller than that of the horseshoe-shaped tunnel. Therefore, the reliability of the circular tunnel increases with the increase of λ, while the reliability of the horseshoe-shaped tunnel decreases accordingly. The difference in vault displacement is determined by the cross-sectional shape, which leads to the opposite trends in the reliability of the two types of tunnels as λ changes. This also confirms the correlation between tunnel reliability and cross-sectional shape.

The mean value of single variable T is controlled from 0 MPa to 1 MPa; the relationship curve between T and reliability is drawn as Fig 6 (c). By comparing the circular tunnel and horseshoe tunnel, it is found that the change rules of the two tunnels are the same, and both increase with the increase of T. Under the same pressure, because the vault displacement of horseshoe tunnel is smaller, the reliability of the horseshoe tunnel is slightly larger than the circular tunnel.

### 4.2. Parameter variability analysis

Building on the research content of the previous section, this section conducts a comparative analysis of the impact of the variability of parameters *E, μ, P, λ*, and *T* on the reliability of horseshoe-shaped tunnels and circular tunnels in accordance with the parameters specified in Table 2. Its core objective is to qualitatively analyze the sensitivity of each parameter to the reliability parameters of tunnels with different cross-sectional shapes.

**Fig 7:**
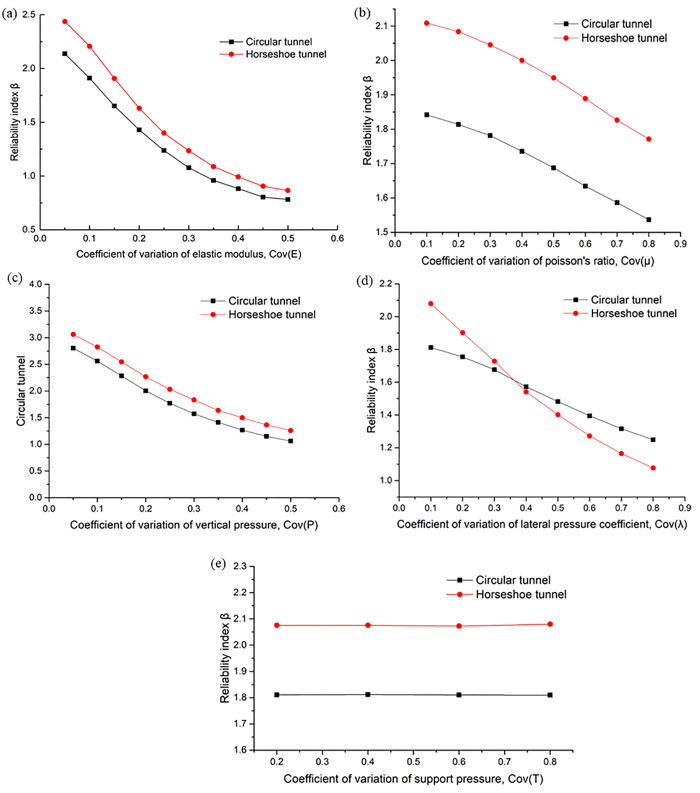
Influence of variation coefficient of parameters on tunnel reliability.

In order to analyze the influence of the variation of E parameter on the reliability of horseshoe tunnel and circular tunnel, the variation coefficient of single variable *E* is controlled from 0.05 to 0.5, the relationship curve between the variation coefficient of *E* and reliability is drawn as Fig 7 (a). It is found that their variation laws are the same, they both decrease with the increase of the variation coefficient of E and the variation range are the same.

As for the variability of the μ parameter, by controlling the variation coefficient of a single variable μ from 0.1 to 0.8, the relationship curve between μ variation coefficient and reliability is drawn as Fig 7 (b). It is shown that the variation law is the same, and both decrease with the increase of Poisson's ratio variation coefficient, and the variation range is not very different.

The variation coefficient of single variable P is uniformly controlled from 0.05 to 0.5, and the relationship curve between the variation coefficient of P and reliability is drawn as shown in Fig 7 (c). It is found that the variation law is the same, and both decrease with the increase of the variation coefficient of P, and the variation range is not very different.

In order to analyze the influence of the variation of λ parameter on the reliability of the horseshoe tunnel and circular tunnel, the variation coefficient of single variable λ was controlled from 0.1 to 0.8. The analysis results are shown in Fig 7 (d). It is found that both of them decrease with the increase of the variation coefficient of λ, but the horseshoe tunnel is more sensitive, the slope is larger, and the variation range is larger.

As for the variability of the T parameter, the variation coefficient of the single variable T was controlled from 0.2 to 0.8. The analysis results are shown in Fig 7 (e). It is shown that both are less affected by the variation coefficient of T and are not sensitive to T.

Through comprehensive analysis Fig 7, it is found that the reliability of horseshoe and circular tunnels vary similarly with parameters, parameter sensitivity from big to small are: P,E,λ,μ,T.

### 4.3. Parameter cross-correlation analysis

There is a certain cross-correlation in geotechnical materials, which have been studied by many scholars [38,39]. In order to analyze the cross-correlation among the parameters, it is necessary to use the Cholesky orthogonal transformation method to convert the variables into independent normal random variables U. In other words, the lower triangular matrix *L* is obtained by using the Joris decomposition $\rho =LL^{T}$ through the correlation coefficient matrix ρ, and the standard normal random variable U can be expressed as $U=L^{T}Y$.

**Fig 8:**
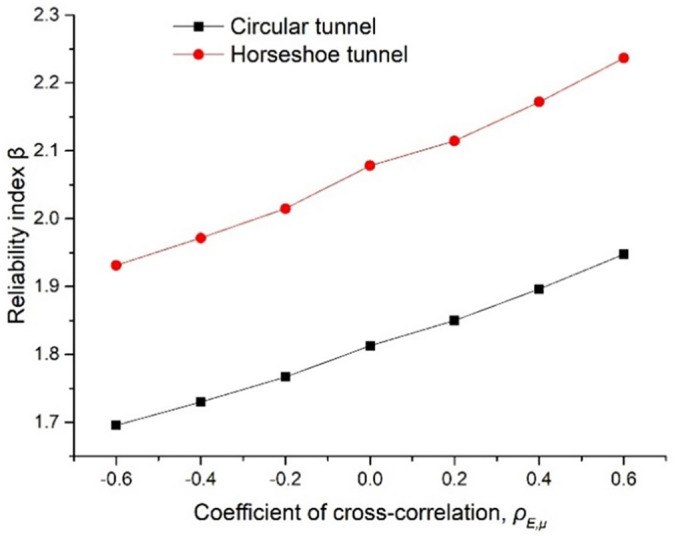
Influence of the correlation coefficient between E and μ on reliability.

The cross-correlation between E and μ of rock mass material and other parameters is shown in Table 2. By controlling the cross-correlation coefficient's uniform variation from −0.6 to 0.6, the relationship curve between the cross-correlation coefficient and reliability is plotted in Fig 8. By comparing the reliability of circular tunnel and horseshoe tunnel, it was found that the reliability of both tunnels increased with the increase of cross-correlation coefficient, and the increased amplitude was consistent. In the same case, the reliability of the horseshoe tunnel is slightly larger.

The research findings can provide theoretical references and engineering insights for the analogous design of horseshoe-shaped tunnels and circular tunnels. However, this study still has several limitations, mainly reflected in the following two aspects: First, the performance function established through analytical derivation is only applicable to scenarios where the surrounding rock is in the elastic stage; Second, the model only selects vault settlement as the core evaluation index and does not cover other key engineering response parameters. The above limitations point out the direction for subsequent research. In the future, the applicable scope of the performance function can be further expanded (e.g., considering the plastic yielding characteristics of the surrounding rock), and multi-dimensional indicators such as surrounding rock convergence, surrounding rock stress, and plastic zone can be incorporated to conduct more comprehensive research on tunnel reliability analysis.

## 5. Conclusions

This study achieves the transformation of a horseshoe-shaped tunnel into a unit circle using the conformal mapping and composite optimization method. Based on the complex variable theory of plane elasticity, an analytical equation for the vault displacement considering support pressure is derived, which is verified to be valid through the ABAQUS numerical solution and the verification under the working condition of degrading to a circular tunnel. Taking this displacement as the limit state, an explicit performance function for tunnel reliability with vault displacement as the indicator is established.

Based on the display function, the results of AFORM, MCS, IS, and the RBIS method was compared and analyzed. By taking 100 million times the MCS method as the exact solution, it was found that the relative error of reliability calculated by the AFORM method was larger, while the IS and RBIS methods were more efficient than the MCS method. In the same case, RBIS showed shorter sampling times and higher computational efficiency than the IS method.

The RBIS method is employed to analyze the influence of load mean value on the reliability of horseshoe-shaped tunnels and circular tunnels in the same area. The results show that: the reliability of both types of tunnels decreases with the increase of average pressure *P* and increases with the increase of support force T; under the same conditions, the horseshoe-shaped tunnel has higher reliability, and its reliability decreases with the increase of lateral pressure coefficient λ, while the reliability of the circular tunnel shows an upward trend with the increase of λ.

The influence law of the coefficient of variation (COV) of load and surrounding rock parameters is explored: the reliability of both the horseshoe-shaped tunnel and the circular tunnel with equal area decreases with the increase of the COV of *E,* μ*, P*, and λ; the support force *T* is not sensitive to the reliability of either tunnel. According to the influence strength, parameters were sorted as follows: P,E,λ,μ,T.

The influence of the cross-correlation coefficient between E and μ on the reliability of the two types of tunnels in the same area is analyzed. It is found that: the reliability of both tunnels increases with the increase of this cross-correlation coefficient, and the magnitude of the increase is consistent; under the same working conditions, the reliability of the horseshoe-shaped tunnel is slightly higher than that of the circular tunnel.

## References

[1] Exadaktylos GE, Stavropoulou MC. A closed-form elastic solution for stresses and displacements around tunnels. International Journal of Rock Mechanics and Mining Sciences. 2002;39(7):905–16. doi: 10.1016/s1365-1609(02)00079-5

[2] Liu Q, Zhang X, Jiang Q, Xia Y, Hou D, Li L. Effects of nano-Al2O3, nano-MgO and nano-Fe2O3 on the properties of cement-based 3D printing: A comparative study. J Build Eng. 2025;113322. doi: 10.1016/j.jobe.2025.113322

[3] Jiang Q, Liu Q, Wu S, Zheng H, Sun W. Modification effect of nanosilica and polypropylene fiber for extrusion-based 3D printing concrete: Printability and mechanical anisotropy. Additive Manufacturing. 2022;56:102944. doi: 10.1016/j.addma.2022.102944

[4] Jiang Q, Liu Q, Wu J, Gong F, Lu X. A review of similar physical simulation in geotechnical engineering: Advances in the past 25 years. Journal of Rock Mechanics and Geotechnical Engineering. 2026. doi: 10.1016/j.jrmge.2025.10.020

[5] Kargar AR, Rahmannejad R, Hajabasi MA. A semi-analytical elastic solution for stress field of lined non-circular tunnels at great depth using complex variable method. International Journal of Solids and Structures. 2014;51(6):1475–82. doi: 10.1016/j.ijsolstr.2013.12.038

[6] Lu A, Zhang N, Qin Y. Analytical solutions for the stress of a lined non-circular tunnel under full-slip contact conditions. International Journal of Rock Mechanics and Mining Sciences. 2015;79:183–92. doi: 10.1016/j.ijrmms.2015.08.008

[7] Guan Z, Deng T, Huang H, Jiang Y. Back analysis technique for mountain tunneling based on the complex variable solution. International Journal of Rock Mechanics and Mining Sciences. 2013;59:15–21. doi: 10.1016/j.ijrmms.2012.11.002

[8] Zeng GS, Wang HN, Jiang MJ, Luo LS. Analytical solution of displacement and stress induced by the sequential excavation of noncircular tunnels in viscoelastic rock. International Journal of Rock Mechanics and Mining Sciences. 2020;134:104429. doi: 10.1016/j.ijrmms.2020.104429

[9] Sousa PFS, Afonso SMB, Willmersdorf RB. Reliability-based preventive maintenance planning for corroded pipelines using a RBF surrogate model. J Braz Soc Mech Sci. 2021;43(12).

[10] Wu WZ, Zhang YX, Liu Y, Hou W, Zhu JX, Jiang F, et al. Impacts of chloride salt, freeze-thaw cycling, and pre-cracking on mechanical properties of Engineered Cementitious Composites (ECC). Constr Build Mater. 2025;475. doi: 10.1016/j.conbuildmat.2025.141221

[11] Zhang Y-X, Gao Y-H, Lin R, Hou W, Yan G-W, Lan J, et al. Tuning superior hydrophobic performance of Engineered/Strain-Hardening Cementitious Composites (ECC/SHCC) via polydimethylsiloxane-based bulk modification. Materials & Design. 2025;254:114117. doi: 10.1016/j.matdes.2025.114117

[12] Mesquita LC, Sotelino ED, Peres ML. Uncertainties consideration in elastically heterogeneous fluid-saturated media using first-order second moment stochastic method and Green’s function approach. Appl Math Model. 2023;115:819–52. doi: 10.1016/j.apm.2022.11.012

[13] Liu X, Yin LR, Hu L, Zhang ZY. An efficient reliability analysis approach for structure based on probability and probability box models. Struct Multidiscip O. 2017;56(1):167–81. doi: 10.1007/s00158-017-1659-7

[14] Breitung K, Hohenbichler M. Asymptotic approximations for multivariate integrals with an application to multinormal probabilities. Journal of Multivariate Analysis. 1989;30(1):80–97. doi: 10.1016/0047-259x(89)90089-4

[15] Hong FQ, Song JW, Wei PF, Huang ZT, Beer M. A stratified beta-sphere sampling method combined with important sampling and active learning for rare event analysis. Struct Saf. 2025;112. doi: 10.1016/j.strusafe.2024.102546

[16] Amirfakhrian M, Mafikandi H. Approximation of parametric curves by Moving Least Squares method. Appl Math Comput. 2016;283:290–8. doi: 10.1016/j.amc.2016.02.039

[17] Safari M, Rabiee AH, Joudaki J. Developing a Support Vector Regression (SVR) Model for Prediction of Main and Lateral Bending Angles in Laser Tube Bending Process. Materials. 2023;16(8). doi: 10.3390/ma16083251 PMC10141193 37110084

[18] Jia J, An S, Tenorio VO. The reliability of cold region tunnels considering deterioration of the insulation layer. PLoS One. 2025;20(4):e0320201. doi: 10.1371/journal.pone.0320201 40184407 PMC11970654

[19] Dai C, Zhou Z, Zhang H, Jiang K, Li H, Yu H. Service reliability evaluation of highway tunnel based on digital image processing. PLoS One. 2023;18(8):e0288633. doi: 10.1371/journal.pone.0288633 37566607 PMC10420349

[20] Lu YX, Lu ZZ. A novel cross-entropy-based importance sampling method for cumulative time-dependent failure probability function. Reliab Eng Syst Safe. 2025;253. doi: 10.1016/j.ress.2024.110511

[21] Zhao Y, Zhang DQ, Yang MD, Wang F, Han X. On efficient time-dependent reliability analysis method through most probable point-oriented Kriging model combined with importance sampling. Struct Multidiscip O. 2024;67(1). doi: 10.1007/s00158-023-03721-7

[22] Liu Q, Jiang Q, Yu Y, Rong Y, Sun Y, Zhao H. Extrusion 3D printing circular and horseshoe tunnel physical models: A comparative study of deformation and brittle failure. Theor Appl Fract Mec. 2024;129:104229. doi: 10.1016/j.tafmec.2023.104229

[23] Napa-Garcia GF, Beck AT, Celestino TB. Reliability analyses of underground openings with the point estimate method. Tunn Undergr Sp Tech. 2017;64:154–63. doi: 10.1016/j.tust.2016.12.010

[24] Guo XF, Du DC, Dias D. Reliability analysis of tunnel lining considering soil spatial variability. Eng Struct. 2019;196.

[25] Lü Q, Xiao ZP, Zheng J, Shang YQ. Probabilistic assessment of tunnel convergence considering spatial variability in rock mass properties using interpolated autocorrelation and response surface method. Geosci Front. 2018;9(6):1619–29. doi: 10.1016/j.gsf.2017.08.007

[26] Zhou SY, Guo XF, Zhang Q, Dias D, Pan QJ. Influence of a weak layer on the tunnel face stability - reliability and sensitivity analysis. Comput Geotech. 2020;122:103507. doi: 10.1016/j.compgeo.2020.103507

[27] Lurie AI. Theory of elasticity. Springer Science & Business Media. 2010.

[28] Muskhelishvili NI. Some basic problems of the mathematical theory of elasticity. Groningen: Noordhoff. 1953.

[29] Huang MK, Jin XG, Wang Y, Zhu L. Studies on influence of tunnel surrounding rock stability analysis based on material yield criterions. Appl Mech Mater. 2011;90–93:501–7. doi: 10.4028/www.scientific.net/AMM.90-93.501

[30] Daraei A, Zare S. A new strain-based criterion for evaluating tunnel stability. Geomech Eng. 2018;16(2):205–15. doi: 10.12989/gae.2018.16.2.205

[31] Chen GH, Zou JF, Qian ZH. Analysis of tunnel face stability with non-linear failure criterion under the pore water pressure. Eur J Environ Civ En. 2020. doi: 10.1080/19648189.2020.1777905

[32] Lopez RH, Beck AT. Reliability-based design optimization strategies based on FORM: A review. J Braz Soc Mech Sci. 2012;34(4):506–14. doi: 10.1590/S1678-58782012000400012

[33] Singh P, Samui P, Mohamad ET, Bhatawdekar RM, Zhang W. Application of MCS, GRNN, and GPR for performing the reliability analysis of rock slope. Nat Hazards. 2024;120(8):7897–917. doi: 10.1007/s11069-024-06472-w

[34] Wu ZH, Chen ZZ, Chen G, Li XK, Jiang C, Gan XH. A probability feasible region enhanced important boundary sampling method for reliability-based design optimization. Structural and Multidisciplinary Optimization. 2021;63(1):341–55. doi: 10.1007/s00158-020-02702-4

[35] Grooteman F. Adaptive radial-based importance sampling method for structural reliability. Structural Safety. 2008;30(6):533–42. doi: 10.1016/j.strusafe.2007.10.002

[36] Xiong B, Tan H. A robust and efficient structural reliability method combining radial-based importance sampling and Kriging. Sci China Technol Sci. 2017;61(5):724–34. doi: 10.1007/s11431-016-9068-1

[37] Wei N, Lu Z, Hu Y. An eccentric radial-based importance sampling method for reliability analysis. Expert Systems with Applications. 2023;219:119687. doi: 10.1016/j.eswa.2023.119687

[38] Jiang SH, Li DQ, Zhang LM, Zhou CB. Slope reliability analysis considering spatially variable shear strength parameters using a non-intrusive stochastic finite element method. Eng Geol. 2014;168:120–8. doi: 10.1016/j.enggeo.2013.11.006

[39] Cheng HZ, Chen J, Chen RP, Chen GL. Comparison of modeling soil parameters using random variables and random fields in reliability analysis of tunnel face. Int J Geomech. 2019;19(1). doi: 10.1061/(asce)gm.1943-5622.0001330

